# Repeated rotation of a toric implantable collamer lens: A case report

**DOI:** 10.1097/MD.0000000000024986

**Published:** 2021-03-12

**Authors:** Haorun Zhang, Mengjun Fu, Jiahao Wang

**Affiliations:** Department of Refractive Surgery Center, Weifang Eye Hospital, Weifang, Shandong, China.

**Keywords:** case report, myopic astigmatism, rotation, toric implantable collamer lens, visual acuity

## Abstract

**Introduction:**

Implantable collamer lens have been used widely worldwide, and have been accepted by more and more doctors and patients due to good safety, stability, and effectiveness. However, there is still a problem of crystal rotation. The large angle rotation (over 10°) would weaken the original astigmatism correction effect and even induce irregular astigmatism, seriously affecting the visual quality of patients. Herein, we reported a case who had 2 times of crystal rotations after toric implantable collamer lens (TICL) implantation.

**Patient concerns:**

The patient was a 38-year-old man who underwent TICL implantation for the correction of high myopic astigmatism in eyes. He presented a sudden decrease in the visual acuity (VA) of the left eye 4 months after the TICL implantation. The uncorrected visual acuity (UCVA) was 8/20 (refraction, +2.25 −5.25 × 68).

**Diagnosis:**

Rotation of TICL was diagnosed. The toric marks with a rotation of 75° counter-clockwise from the original position were observed.

**Interventions:**

The TICL was re-set to the original position, leading to the UCVA of 12/20 in the left eye (refraction, −0.00 −0.75 × 131), with the vaulting of 589 μm. Ten months after the TICL relocation, the patient again presented a sudden decrease in the VA of the left eye, with the UCVA of 2/20 (refraction, +2.25 −5.00 × 66). Again, the toric marks with a rotation of 75° counter-clockwise from the original position was observed, just at the same position as the last rotation. This time, the TICL was removed. The axis and power were recalculated, and a new TICL was implanted, with the rotation of 73° counter-clockwise from the horizontal line of the temporal side.

**Outcomes:**

The patient obtained a final UCVA of 12/20 in the left eye (refraction, +0.50 −0.50 × 26), which remained stable in the 6-month follow-up period, with the vaulting of 602 μm.

**Lessons:**

Rotation is a common complication after TICL surgery. Relocation or replacement of TICL are safe and efficient ways to recover VA due to TICL rotation.

## 1 Introduction

Toric implantable collamer lens (TICL) has good safety, stability, and effectiveness, but there is still a problem of crystal rotation. Axial rotation within 10° will not lead to serious visual function decline. However, the large angle rotation (over 10°) would weaken the original astigmatism correction effect and even induce irregular astigmatism, seriously affecting the visual function of patients. If the TICL rotation reaches 30°, the degree of astigmatism used for correction will be completely offset. The rotation of TICL crystals is mainly related to the structure of the ciliary sulci and the size of the crystals. The process of crystal rotation is to adjust or replace the crystal. Adjustment and replacement of the lens will not cause much damage to the eye, but can also achieve more desirable results. Herein, we reported a case who had 2 times of crystal rotations. For the first rotation of the crystal, crystal adjustment was performed. Postoperative vision and arch height were ideal. For the second rotation of the crystal, we decided to replace the crystal with the near-vertical position to achieve the desired effect. The crystal position, arch height, and visual acuity were stable after operation.

## 2 Case report

This study was approved by the Ethics Committee of Weifang Eye Hospital. Written informed consent was obtained from the patient.

**Figure 1:**
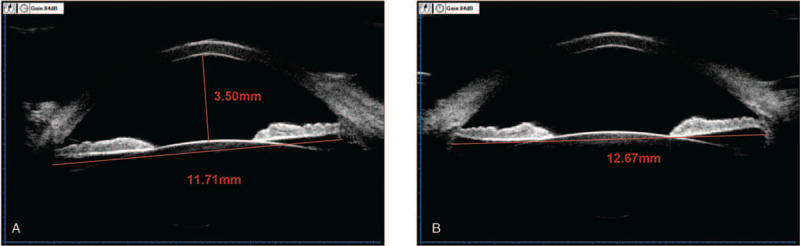
UBM measurement of ACD and STS of the left eye. (A) ACD was 3.50 mm and the horizontal (0°–180°) STS was 11.71 mm. (B) The vertical (90°–270°) STS was 12.67 mm. ACD = anterior chamber depth, STS = sulcus-to-sulcus, UBM = ultrasound biomicroscopy.

**Figure 2:**
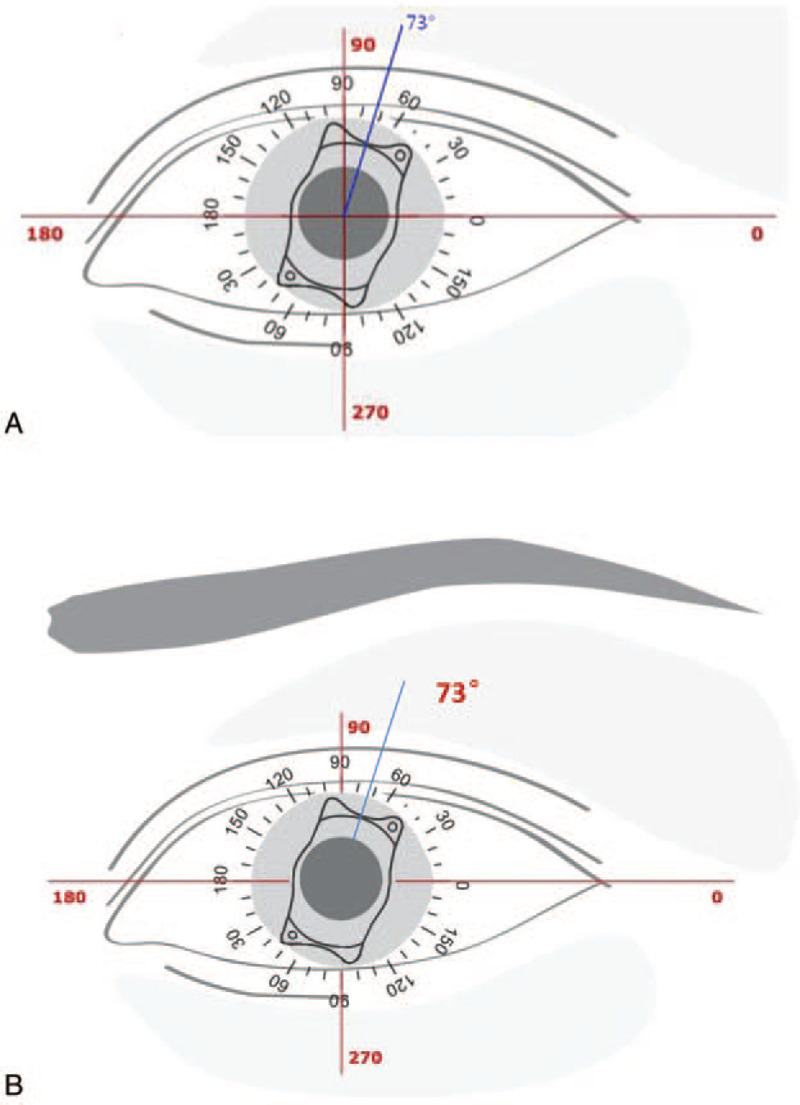
TICL calculation and location of the left eye. (A) The TICL was supposed to rotate with 2° clockwise from the horizontal line of the temporal side. (B) The new TICL was supposed to rotate with 73° counter-clockwise from the horizontal line of the temporal side. TICL = Toric implantable collamer lens.

Herein, we reported a case of a 38-year-old man who underwent the TICL implantation for the correction of high myopic astigmatism in eyes. Before operation, the patient's uncorrected visual acuities (UCVA) were 0.4/20 for both eyes. The corrected distant visual acuity was 14/20 in the right eye (refraction, −10.75 −4.00 × 90) and 14/20 in the left eye (refraction, −11.25 −3.75 × 75). Moreover, the IOP was 14-mm Hg for both eyes. The central corneal thicknesses were 518 μm in the right eye and 508 μm in the left eye. The white-to-white diameter was 11.1 mm in the right eye and 11.2 mm in the left eye (Pentacam; Oculus, Wetzlar, Germany). The endothelial cell density was 2601.2/mm^2^ in the right eye and 2524.8/mm^2^ in the left eye. Ultrasound biomicroscopy (UBM; Quantel Medical, France) revealed that the anterior chamber depth was 3.50 mm for both eyes. The horizontal (0°–180°) ciliary sulcus-to-sulcus (STS) was 11.35 mm in the right eye and 11.71 mm in the left eye (Fig. 1A). The vertical (90°–270°) STS was 12.39 mm in the right eye and 12.67 mm in the left eye (Fig. 1B). The ocular axial length was 29.84 mm in the right eye and 30.17 mm in the left eye (IOL Master 700; Carl Zeiss Meditec AG, Jena, Germany). The ICL power was calculated using the modified vertex formulation provided by the manufacturer (STAAR Surgical, Nidau, Switzerland). The patient received a 12.1-mm-long TICL in each eye: −14.5/+3.5/180 in the right eye, with horizontal implantation and −15.0/+3.5/167 in the left eye, with rotation (2° clockwise) from the horizontal implantation (Fig. 2). The surgical procedure was as follows: an eyelid opener was placed, and 0.5% proparacaine hydrochloride eye drop (Alcaine) was applied 2 times. A 2.6-mm scalpel was used to make an incision at 130° along the corneal margin, and the viscoelastic agent (1.7% sodium hyaluronate; Bausch & Lomb, China) was injected into the eyes. Then the TICL V4c was inserted into each eye through the incision using an injector cartridge (STAAR Surgical), which was adjusted to the suitable position in the posterior chamber. The viscoelastic agent was completely removed with the balanced salt solution. After the surgical operation, the patient was treated with 1% prednisolone acetate, three times a day, for 1 week, as well as Vigamox, 3 times a day, for 1 week. After operation, he achieved the UCVA of 14/20 in the right eye and 16/20 in the left eye. The vault was 547 μm in the right eye and 563 μm in the left eye (Visante OCT; Carl Zeiss Meditec AG).

**Figure 3:**
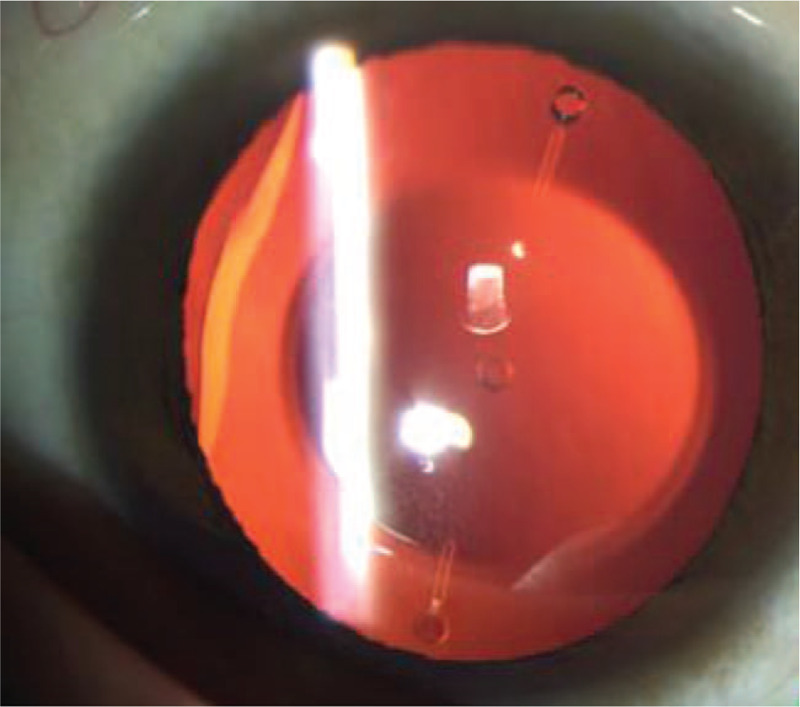
Anterior segment photograph of the left eye. The toric marks rotated 75° counter-clockwise from the original position.

At four months after the TICL implantation, the patient presented a sudden decrease in the VA of the left eye, with the UCVA of 8/20 (refraction, +2.25 −5.25 × 68). The toric marks with a rotation of 75° counter-clockwise from the original position were then observed (Fig. 3). Therefore, the TICL was re-set to the original position, leading to the UCVA of 12/20 in the left eye (refraction, −0.00 −0.75 × 131), with the vaulting of 589 μm.

**Figure 4:**
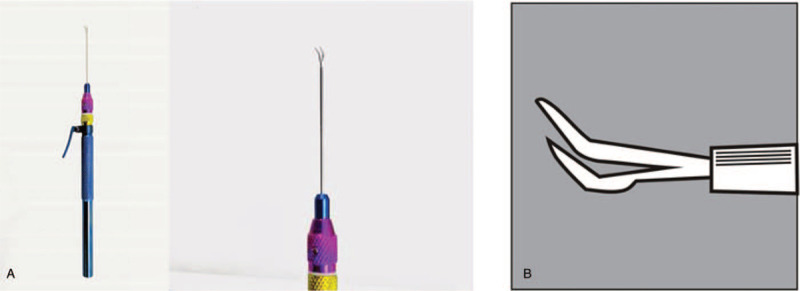
Tweezer used to remove the TICL. The tweezer was straight shafts angled type tips, 20 G/0.9 mm, with round knurled handles and overall length of 150 mm, made of titanium and polished. (A) Photo of tweezer; (B) The tweezer head. TICL = Toric implantable collamer lens.

At 10 months after the TICL relocation, the patient again presented a sudden decrease in the VA of the left eye, with the UCVA of 2/20 (refraction, +2.25 −5.00 × 66). Again, the toric marks with a rotation of 75° counter-clockwise from the original position was observed, just at the same position as the last rotation. This time, the TICL was removed. The axis and power were recalculated, and the new TICL was implanted, with the rotation of 73° counter-clockwise from the horizontal line of the temporal side (Fig. 2). The surgical procedures were as follows: the eyelid opener was placed, and a 2.6-mm incision was made from the original position. The viscoelastic agent was injected into the anterior chamber. A hook was used to pick out the TICL footplate. The TICL was clamped and removed away with a tweezer (Fig. 4). The viscoelastic agent was then completely removed with the balanced salt solution. Thereafter, the new TICL was implanted into the eye, which was adjusted to the suitable position. The patient was subjected to the post-operation treatment as described above. After the implantation, the patient obtained a final UCVA of 12/20 in the left eye (refraction, +0.50 −0.50 × 26), which remained stable in the 6-month follow-up period, with the vaulting of 602 μm.

## 3 Discussion

ICL is a posterior chamber phakic IOL, which has been approved by FDA in December 2005. Recently, the TICL has been demonstrated to be effective and safe in correcting the high myopic astigmatism, in comparison with the laser corneal surgery, with significantly improved contrast sensitivity function.^[1–3]^ The optical region of the TICL crystal is 4.65 to 5.5 mm in diameter, which can correct the astigmatism of 0.50 to 6.00 D. However, the premise for obtaining good outcomes is the accurate and stable position of TICL in the eye. The TICL rotation would lead to the decreased or disappearance of astigmatism correction and the patient's dissatisfaction. Axial rotation is a relatively common phenomenon after TICL surgery. Axial rotation within 10° will not lead to serious visual function decline,^[4]^ and, however, the large angle rotation (over 10°) would weaken the original astigmatism correction effect and even induce irregular astigmatism, seriously affecting the visual function of prognosis. If the TICL rotation reaches 30°, the degree of astigmatism used for correction will be completely offset. When the TICL rotation exceeds 30°, postoperative astigmatism will be increased.^[5]^ In patients with high astigmatism, axial deviation caused by postoperative TICL rotation will further lead to oblique astigmatism and obvious uncomfortable symptoms.

Several factors can cause the rotation after TICL implantation. First, TICL rotation has a certain correlation with intraoperative lens implantation angle and preoperative astigmatism degree. For patients with high astigmatism, keeping the rotation angle within 10° during TICL implantation can effectively reduce the postoperative rotation and increase the stability.^[6]^ Second, the TICL footplate-position and vault value should be taken into consideration, as two possible risk factors for TICL rotation. A previous study has shown that UBM-revealed footplates of TICL are in the ciliary sulcus in 22 eyes (46.3%) and below the ciliary sulcus in 32 eyes (53.7%). As a result, TICL rotation has been observed in 20 out of the 22 eyes (90.9%) with footplates fixed in the ciliary sulcus and in 14 out of the 32 eyes (43.8%) with footplate fixed below the ciliary sulcus.^[7]^ Furthermore, the angle of TICL rotation has significant correlation with the diameter and structure of the ciliary STS. Previous findings have shown that, in 35 out of 37 eyes, 90° STS (12.51 ± 0.43 mm) is greater than 0° STS (12.19 ± 0.47 mm).^[8]^ In this study, the horizontal STS was 11.71 mm and the vertical STS was 12.67 mm. We believed that the spontaneous rotation of TICL might be related to the diameter and structure of the ciliary STS. After reviewing our case, we decided to replace the TICL and place it in the used rotation position. However, the STAAR Surgical TICL calculator could only allow the maximum rotation angle of 22°, and when calculating the crystal, the axial position of the column mirror would be changed by 90°, while other calculated data remained unchanged. We designed to implant the TICL crystal into the ideal position to achieve the satisfactory. After the TICL crystal implantation, the patient was satisfied, who was followed up for 6 months, during which the TICL position was stable.

Secondary transposition is necessary for patients with large axial rotation accompanied by severe visual impairment and/or visual quality decline. We believe that the indications for the secondary transposition include:

(1) the rotation of TICL exceeding 10°;
(2) UCVA of TICL implantation lower than BCVA; and
(3) patients with subjective visual quality questionnaire.^[9]^

For the secondary transposition in the case reported herein, the following factors needed to be considered:

(1) determining the footplate position of TICL crystal by UBM;
(2) determining the diameter and structure of ciliary STS by UBM; and
(3) if the ICL crystal rotation angle was relatively large, which needed to be placed in the near-vertical position, the TICL needed to be recalculated by the axial position of the column mirror changed by 90°, with other calculated data unchanged.

Sometimes a larger size would be necessary, according to diameter of ciliary STS. During the operation, we could sit on the temporal side of the patient, and mark according to the given axial position. The tweezer could be used when the ICL was taken out.

## 4 Conclusions

In conclusion, our results showed that TICL would be a safe, effective and stable way to correct the high myopia astigmatism. Previous studies had indicated the stability of TICL in the eyes.^[10,11]^ The mean deviation of TICL axis, from one week to six months postoperation, is 2.48° ± 1.25° (ranging from 1° to 6°).^[12]^ At 2 years after the operation, 68.5% would have a rotation degree less than 5°, indicating good TICL stability in the eye.^[13]^ Axis rotation after surgery may result in the deterioration of visual performance, consequently inducing patient's dissatisfaction. Relocation and replacement of TICL would be safe and effective methods to cure the spontaneous rotation.

## Author contributions

**Data curation:** Mengjun Fu, Jiahao Wang.

**Methodology:** Haorun Zhang.

**Project administration:** Mengjun Fu.

**Resources:** Mengjun Fu.

**Supervision:** Haorun Zhang, Mengjun Fu.

**Writing – original draft:** Mengjun Fu.
